# Problematic alcohol use and its impact on liver disease quality of life in a multicenter study of patients with cirrhosis

**DOI:** 10.1097/HC9.0000000000000379

**Published:** 2024-02-03

**Authors:** Jeremy W. Luk, Derek D. Satre, Ramsey Cheung, Robert J. Wong, Alexander Monto, Jennifer Y. Chen, Steven L. Batki, Michael J. Ostacher, Hannah R. Snyder, Amy M. Shui, Meimei Liao, Christina G. Haight, Mandana Khalili

**Affiliations:** 1 Office of the Clinical Director, National Institute on Alcohol Abuse and Alcoholism, Bethesda, Maryland, USA; 2 Department of Psychiatry and Behavioral Sciences, Weill Institute for Neurosciences, University of California, San Francisco, California, USA; 3 Kaiser Permanente Northern California, Division of Research, Oakland, California, USA; 4 Department of Medicine, Division of Gastroenterology and Hepatology, Stanford University School of Medicine, Stanford, California, USA; 5 Gastroenterology Section, Veterans Affairs Palo Alto Health Care System, Palo Alto, California, USA; 6 Division of Gastroenterology and Hepatology, Veterans Affairs San Francisco Health Care System, San Francisco, California, USA; 7 Department of Medicine, University of California, San Francisco, San Francisco, California, USA; 8 Liver Center, University of California, San Francisco, San Francisco, California, USA; 9 Department of Medicine, Division of Gastroenterology and Hepatology, Zuckerberg San Francisco General, San Francisco, California, USA; 10 Mental Health Service, Veterans Affairs San Francisco Health Care System, San Francisco, California, USA; 11 Department of Psychiatry and Behavioral Sciences, Stanford University School of Medicine, Stanford, California, USA; 12 Department of Psychiatry, Veterans Affairs Palo Alto Health Care System, Palo Alto, California, USA; 13 Department of Family and Community Medicine, University of California, San Francisco, California, USA; 14 Department of Epidemiology and Biostatistics, University of California, San Francisco, San Francisco, California, USA

## Abstract

**Background:**

Management of cirrhosis is challenging and has been complicated by the COVID-19 pandemic due to decreased access to care, increased psychological distress, and alcohol misuse. Recently, The National Institute on Alcohol Abuse and Alcoholism has broadened the definition of recovery from alcohol use disorder to include quality of life (QoL) as an indicator of recovery. This study examined the associations of alcohol-associated cirrhosis etiology and problematic drinking with liver disease QoL (LDQoL).

**Methods:**

Patients with cirrhosis (N=329) were recruited from 3 sites (63% from 2 Veterans Affairs Health Care Systems and 37% from 1 safety net hospital) serving populations that are economically or socially marginalized. Cirrhosis etiology was ascertained by chart review of medical records. Problematic drinking was defined by ≥8 on the Alcohol Use Disorders Identification Test. Multivariable general linear modeling adjusting for age, sex, race/ethnicity, site, pandemic-related stress, and history of anxiety/depressive disorder were conducted. Sensitivity analyses further adjusted for indicators of liver disease severity.

**Results:**

Participants were on average 64.6 years old, 17% female, 58% non-White, 44% with alcohol-associated cirrhosis, and 17% with problematic drinking. Problematic drinking was significantly associated with worse LDQoL scores in the overall scale and in the memory/concentration and health distress subscales. These associations remained significant after adjusting for indicators of liver disease severity, including Model for End-Stage Liver Disease-Sodium score and decompensated cirrhosis status.

**Conclusions:**

Among patients with cirrhosis, problematic drinking was associated with worse LDQoL, especially in the domains of memory/concentration and health distress. Assessment and awareness of cognitive deficits and negative emotionality within the context of cirrhosis and problematic drinking may help clinicians provide better integrated care for this population.

## Graphical abstract

**Figure:**
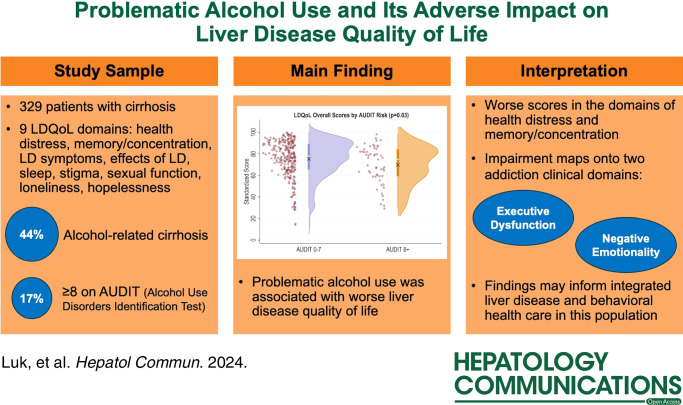

## INTRODUCTION

Alcohol use disorder (AUD) and alcohol-associated liver disease (ALD) are serious chronic conditions associated with increased disability and premature mortality.^1^ Before the COVID-19 pandemic, it was projected that age-standardized deaths due to ALD in the United States would increase from 8.23 per 100,000 person-years in 2019 to 15.20 per 100,000 person-years in 2040.^2^ The COVID-19 pandemic has contributed to the worsening of chronic liver disease and ALD through delays in care, psychological strain, and increased alcohol consumption.^3^ Compared to before the pandemic, patients with ALD who were hospitalized during the COVID-19 pandemic had worse clinical outcomes.^4^ In a modeling study, the 1-year increase in alcohol consumption during the first year of the COVID-19 pandemic was estimated to result in 8000 more ALD-related deaths, 18,700 more cases of decompensated cirrhosis, and a loss of 8.9 million disability-adjusted life years in the next 2 decades.^5^ These studies highlight the public health relevance of studying problematic drinking and ALD in the context of the COVID-19 pandemic, especially among socioeconomically and medically disadvantaged populations disproportionally at risk for adverse health outcomes.

Given the psychological distress associated with the initial lockdowns, researchers predicted increased alcohol use and misuse as a coping mechanism during the pandemic.^6,7^ According to a meta-analysis, 16% of the individuals in the United States decreased alcohol consumption during the pandemic, whereas 29% increased alcohol consumption.^8^ Individuals with AUD have been identified as a group disproportionately affected by the COVID-19 pandemic, as interruptions to treatment and recovery support networks may precipitate relapse and lead to alcohol-associated emergencies and other medical complications.^9,10^ Moreover, pandemic-related stressors were associated with increased drunkenness frequency among individuals with a history of AUD.^11^ As pandemic-related alcohol consumption could lead to long-term adverse health outcomes,^12^ ensuring access to quality treatment of AUD and ALD remains an important public health priority in the transition to the COVID-19 endemic phase.^13^

To address ALD and AUD in a comprehensive way, the promotion of quality of life (QoL) has been increasingly recognized as a key part of holistic treatment.^14^ Measurement and promotion of patient-reported outcomes including QoL are considered a critical part of patient-centered clinical care for cirrhosis.^15^ The National Institute on Alcohol Abuse and Alcoholism (NIAAA) has recently broadened its research definition of AUD recovery to include measures of biopsychosocial well-being and QoL as clinical outcomes.^16^ There is a robust literature showing inverse associations between drinking levels and QoL domains, including physical, psychological, social, and environment as defined by the World Health Organization.^17,18^ Focusing on these general QoL domains not tied to a specific disease, studies conducted during the COVID-19 pandemic showed that individuals with a history of AUD may have lower QoL than those without AUD to begin with,^19^ and that COVID-related stressors may have worsened QoL during the pandemic.^20^ These associations may persist and have lasting effects in the postpandemic era.

Several measures of liver disease–specific QoL have been developed and can be used to capture health-related QoL among patients with chronic liver disease.^21^ However, despite the availability of these measures, the extent to which ALD etiology and problematic drinking are associated with liver disease QoL domains remains understudied, especially among patients with cirrhosis. Therefore, the aim of this study was to examine the associations of alcohol-associated cirrhosis etiology and problematic drinking based on the Alcohol Use Disorders Identification Test (AUDIT) with liver disease QoL in a diverse sample of patients with cirrhosis. We hypothesized that compared to participants with nonalcohol cirrhosis etiology, participants with alcohol cirrhosis etiology would report lower liver disease quality of life (LDQoL) scores. We also hypothesized that compared to the participants with AUDIT <8, the participants with AUDIT ≥8 (indicative of problematic drinking) would report lower LDQoL scores.

## METHODS

### Study participants and data collection

**FIGURE 1:**
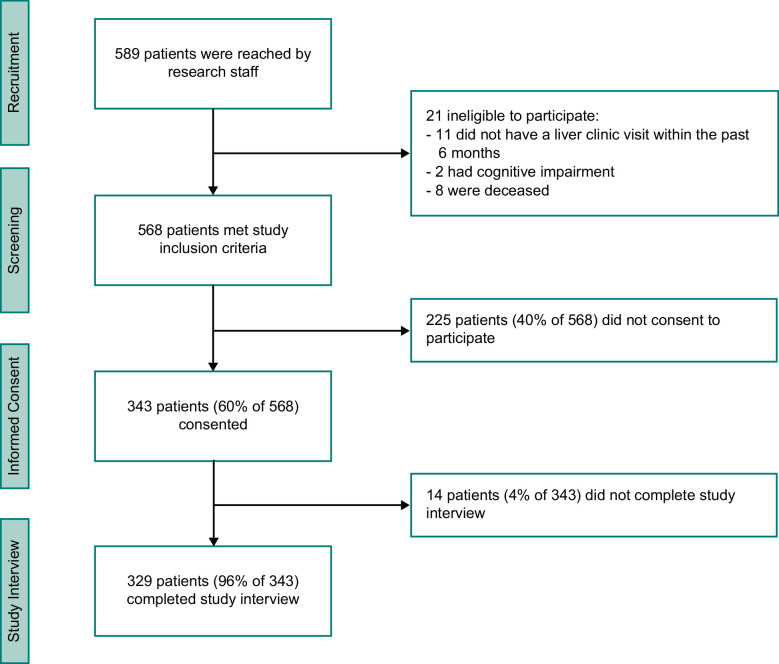
Of the patients who met study inclusion criteria, 60% gave informed consent. Among those who gave informed consent, 96% completed the study interview.

Patients receiving cirrhosis care were identified using electronic medical record data from 3 medical centers in Northern California: Palo Alto Veterans Affairs Health Care System (PAVA), San Francisco Veterans Affairs Health Care System (SFVA), and Zuckerberg San Francisco General Hospital (ZSFG), a hospital affiliated with the San Francisco safety-net health system. The diagnosis of cirrhosis by a liver specialist was further verified by review of hepatology clinic notes. Patients who received clinical services at these 3 medical centers are often vulnerable to poor outcomes due to medical complexity, low socioeconomic status, and limited resources. Eligibility criteria included being age 18 or older, English-speaking or Spanish-speaking, a clinical diagnosis of cirrhosis, and at least 1 hepatology clinic visit within the prior 6 months. Patients unwilling or unable to give informed consent, for example, due to diminished cognitive capacity, were excluded. Eligible patients were contacted through mail, telephone, and in person during clinic visits. They were invited to participate in a one-time survey on patient-reported symptoms and QoL measures, which was administered by trained research personnel over the phone or in person. Interviews with Spanish-speaking participants were assisted by certified medical interpreters on an as-needed basis. Figure 1 shows participant flow from recruitment, screening, and informed consent to study interview completion. All participants provided informed consent and were compensated with $35 for their participation. This study was approved by the Institutional Review Boards of the University of California, San Francisco, Stanford University, and by local review committees at PAVA, SFVA, and ZSFG.

### Measures

#### Liver disease quality of life

Participants completed the Short Form of the LDQoL instrument, which was developed based on the SF-36 with modifications to tailor the items to capture liver disease–specific QoL domains.^22^ The Short Form LDQoL instrument has been prospectively validated among patients with advanced liver disease^23^ and has 9 subscales measuring LDQoL domains: symptoms of liver disease, effects of liver disease, memory/concentration, health distress, sexual function, sleep, loneliness, hopelessness, and stigma. An overall score along with 9 subscale scores were calculated and standardized for analysis. These scores were scaled from 0 to 100 with higher overall and subscale scores indicating better LDQoL.

#### Cirrhosis diagnosis and etiology

Cirrhosis diagnosis and etiology were ascertained by chart review of electronic medical records including hepatology clinic notes using standard operating procedures developed across sites. All participants had a clinical diagnosis of cirrhosis by a liver specialist as recorded in their hepatology clinic note. Participants were categorized into 2 groups based on cirrhosis etiology: ALD (44%) versus non-ALD (56%). Some patients had coexisting etiologies. Among the patients with ALD (n=145), 26% had coexisting hepatitis C, 18% had metabolic dysfunction–associated steatotic liver disease, 2.8% had hepatitis B, and 2.8% had other etiologies. Among the patients with no ALD (n=184), the etiologies of cirrhosis were 52% hepatitis C, 30% metabolic dysfunction–associated steatotic liver disease, 9.2% hepatitis B, and 8.7% other etiologies.

#### Cirrhosis severity

Decompensated cirrhosis was defined based on hepatology clinical note documentation and the presence of any of the following decompensation clinical events: ascites, variceal bleeding, encephalopathy, or Child-Pugh Class B or C.^24^ The Model for End-Stage Liver Disease-Sodium (MELD-Na) score was calculated based on the laboratory measures obtained within the prior 6 months.^25^

#### Problematic drinking

The AUDIT was developed by the World Health Organization as a screening tool for hazardous alcohol use in primary health care settings.^26^ A cutoff of 8 or above is a well-established clinical threshold that indicates harmful alcohol use^27^ and was used to categorize participants into 2 groups: problematic drinking (AUDIT ≥8; 17%) and subthreshold or no alcohol use (AUDIT <8; 83%).

#### Pandemic-related stress

COVID-19 pandemic–related stress was measured using an item from the Pandemic Stress Index: “How much is/did COVID-19 (coronavirus) impact your day-to-day life?”^28^ Participants rated the impact of COVID-19 on a Likert response scale that ranged from 1 (not at all) to 5 (extremely). A higher rating indicated higher pandemic-related stress.

#### Anxiety and depression

History of anxiety disorder and major depressive disorder was collected from medical records. In addition, the Generalized Anxiety Disorder-7 (GAD-7) and Patient Health Quesitonnaire-8 (PHQ-8) were utilized to capture current symptoms of anxiety and depression, and a clinical threshold of 10 was used to indicate probable anxiety disorder and probable depressive disorder, respectively.^29,30^

#### Demographic and other measures

Participants self-reported their age, sex, race/ethnicity, household size, annual household income, education level, employment status, marital status, born inside/outside of the United States, English fluency, and any past-year alcohol use.

### Statistical analyses

Statistical analyses were conducted in 4 steps. First, differences in demographic characteristics by cirrhosis etiology (ALD vs. non-ALD) and by problematic drinking (AUDIT ≥8 vs. <8) were compared using *t* tests and chi-square tests. Second, mean differences in the overall LDQoL score and LDQoL subscale scores were compared based on cirrhosis etiology and problematic drinking groups using *t* tests. Third, multivariable general linear models adjusting for age, sex, race/ethnicity, site, pandemic-related stress, and history of anxiety or depressive disorder were conducted. Finally, in a subsample of 310 participants with available data on both MELD-Na and decompensated cirrhosis status, sensitivity analyses were conducted to examine if the observed associations would remain significant after adjusting for these 2 variables as additional covariates. Linear regression assumptions were assessed using quantile-quantile and residual plots. Hypothesis tests were two-sided, and the significance threshold was set to 0.05. Statistical analyses were conducted in SAS 9.4 (SAS Institute).

## RESULTS

Demographic characteristics for the overall sample and differences by cirrhosis etiology and problematic drinking are presented in Table 1. The mean age of participants was 64.6 years old (SD=10.6). The study sample was 83% male and was diverse in terms of race/ethnicity (42% White, 33% Latino, and 25% other groups combined). Participants came from 3 sites with 34% from PAVA, 29% from SFVA, and 37% from ZSFG. The most common household size was living with 1–2 people (including self) (69%), and 55% had household income ≤$30,000. Most participants had high school or more education (80%) and spoke English well or fluently (85%). About one quarter of participants were born outside of the United States (26%). Paid employment (full time or part time) was reported by 23% of the participants. Being married/partnered was reported by 30% of the participants. Overall, 64% of the participants had compensated cirrhosis, and only 20% had current cirrhosis decompensation. The mean MELD-Na score was 10.9 (SD=4.4) and the median was 9 (interquartile range=8–13).

**TABLE 1:** Demographic characteristics for the overall sample and by cirrhosis etiology and problematic drinking status

		Cirrhosis etiology			Problematic drinking		
	Overall	ALD (n=145; 44%)	Non-ALD (n=184; 56%)		AUDIT<8 (n=274; 83%)	AUDIT≥8 (n=55; 17%)	
Continuous variables	M (SD)	M (SD)	M (SD)	*p*	M (SD)	M (SD)	*p*
Age (y)	64.6 (10.6)	61.2 (11.2)	67.2 (9.3)	<0.001	65.5 (10.3)	59.9 (11.1)	<0.001
Pandemic-related stress (points)	2.7 (1.3)	2.8 (1.3)	2.6 (1.3)	0.26	2.7 (1.3)	2.7 (1.3)	0.92
MELD-Na Score^a^	10.9 (4.4)	11.4 (4.7)	10.5 (4.1)	0.09	10.8 (4.3)	11.4 (4.9)	0.34
Categorical variables	*n* (%)	*n* (%)	*n* (%)	*p*	*n* (%)	*n* (%)	*p*
Male sex	272 (83)	130 (90)	142 (77)	0.003	220 (80)	52 (95)	0.01
Race/ethnicity^b^							
White/Caucasian	138 (42)	52 (36)	86 (47)	0.002	118 (43)	20 (36)	0.71
Latino	109 (33)	59 (41)	50 (27)		89 (32)	20 (36)	
Black/African American	31 (9.4)	13 (9.0)	18 (9.8)		24 (8.8)	7 (13)	
Asian/Pacific Islander	26 (7.9)	5 (3.4)	21 (11)		22 (8.0)	4 (7.3)	
Native American	6 (1.8)	5 (3.4)	1 (0.5)		6 (2.2)	0 (0.0)	
Other	19 (5.8)	11 (7.6)	8 (4.3)		15 (5.5)	4 (7.3)	
Site							
PAVA	111 (34)	56 (39)	55 (30)	0.03	90 (33)	21 (38)	0.75
SFVA	97 (29)	32 (22)	65 (35)		82 (30)	15 (27)	
ZSFG	121 (37)	57 (39)	64 (35)		102 (37)	19 (35)	
Household size^c^							
Live with 1–2 people	228 (69)	104 (72)	124 (67)	0.15	188 (69)	40 (73)	0.64
Live with 2–5 people	80 (24)	36 (25)	44 (24)		67 (24)	13 (24)	
Live with >5 people	21 (6.4)	5 (3.4)	16 (8.7)		19 (6.9)	2 (3.6)	
Household income^d^							
≤$30,000	122 (55)	52 (55)	70 (56)	0.85	98 (54)	24 (65)	0.21
Education level							
High school or more	264 (80)	121 (83)	143 (78)	0.19	216 (79)	48 (87)	0.15
Paid employment	77 (23)	44 (30)	33 (18)	0.008	60 (22)	17 (31)	0.15
Married/Partnered	100 (30)	36 (25)	64 (35)	0.05	90 (33)	10 (18)	0.03
Born Outside of US	84 (26)	35 (24)	49 (27)	0.61	70 (26)	14 (25)	0.99
Speaks English well or fluently^e^	280 (85)	128 (88)	152 (83)	0.15	230 (84)	50 (91)	0.19
Coexisting etiologies							
Hepatitis B	21 (6.4)	4 (2.8)	17 (9.2)	0.02	20 (7)	1 (2)	0.13
Hepatitis C	133 (40)	38 (26)	95 (52)	<0.001	119 (43)	14 (25)	0.01
MASLD^f^	82 (25)	26 (18)	56 (30)	0.01	76 (28)	6 (11)	0.01
Other etiologies	20 (6.1)	4 (2.8)	16 (8.7)	0.03	20 (7)	0 (0)	0.04
Decompensated cirrhosis^g^							
Current	62 (20)	42 (29)	20 (12)	<0.001	50 (19)	12 (22)	0.87
Prior	52 (17)	39 (27)	13 (7.6)		43 (17)	9 (17)	
None	200 (64)	63 (44)	137 (81)		167 (64)	33 (61)	
History of anxiety disorder	26 (7.9)	14 (10)	12 (6.5)	0.30	21 (7.7)	5 (9.1)	0.72
History of major depressive disorder	69 (21)	34 (23)	35 (19)	0.33	58 (21)	11 (20)	0.85
Probable anxiety (GAD-7≥10)	46 (14)	27 (19)	19 (10)	0.03	38 (14)	8 (15)	0.89
Probable depression (PHQ-8≥10)	73 (22)	35 (24)	38 (21)	0.45	58 (21)	15 (27)	0.32
Past-year alcohol use^h^	129 (39)	73 (51)	56 (30)	<0.001	74 (27)	55 (100)	<0.001
Problematic drinking (AUDIT≥8)	55 (17)	51 (35)	4 (2.2)	<0.001	NA	NA	

a Four participants had missing data on MELD-Na score.

b Race was coded as White, Latino, and Other for statistical analyses.

c Household size includes oneself.

d One hundred and nine participants had missing data on household income.

e Response options for English fluency included fluent like a native English speaker, speaking English well, so-so, poorly, or not at all.

f Of the 82 patients with MASLD, 12 (15%) also had hepatitis C.

g Fifteen participants had missing data on decompensated cirrhosis status.

h One participant had missing data on any past-year alcohol use.

Abbreviations: ALD, alcohol-associated liver disease; AUDIT, Alcohol Use Disorders Identification Test; GAD-7, Generalized Anxiety Disorder-7; MASLD, metabolic dysfunction–associated steatotic liver disease; MELD-Na, Model for End-Stage Liver Disease-Sodium; NA, Not Applicable; PAVA, Palo Alto Veterans Affairs Health Care System; PHQ-8, Patient Health Quesitonnaire-8; SFVA, San Francisco Veterans Affairs Health Care System; ZSFG, Zuckerberg San Francisco General Hospital.

Compared to participants with non-ALD etiology, participants with ALD cirrhosis were younger (*p*<0.001) and were more likely to be male (*p*=0.003), and had paid employment (*p*=0.008). The distributions of race/ethnicity (*p*=0.002) and site (*p*=0.03) also differed between the 2 groups, with higher percentages of Latino participants (41% vs. 27%) and participants from PAVA (39% vs. 30%) in the ALD cirrhosis group compared to the participants in the non-ALD cirrhosis group. History of anxiety disorder (8% in the overall sample), history of depressive disorders (21% of the overall sample), and probable depressive disorder (22% of the overall sample) did not statistically differ by ALD etiology or problematic drinking. Probable anxiety disorder was higher in the ALD cirrhosis group than in the non-ALD cirrhosis group (19% vs. 10%, *p*= 0.03) but did not statistically differ by problematic drinking. Proportion of AUDIT ≥8 was higher among those with ALD than among those with non-ALD liver disease etiology (35% vs. 2%, *p*<0.001). Compared to those with AUDIT <8, participants with AUDIT ≥8 were younger (*p*<0.001), more likely to be male (*p* = 0.01), and less likely to be married/partnered (*p*=0.03). Past-year alcohol use was reported by 51% of those with ALD and by 30% of those with non-ALD.

**FIGURE 2:**
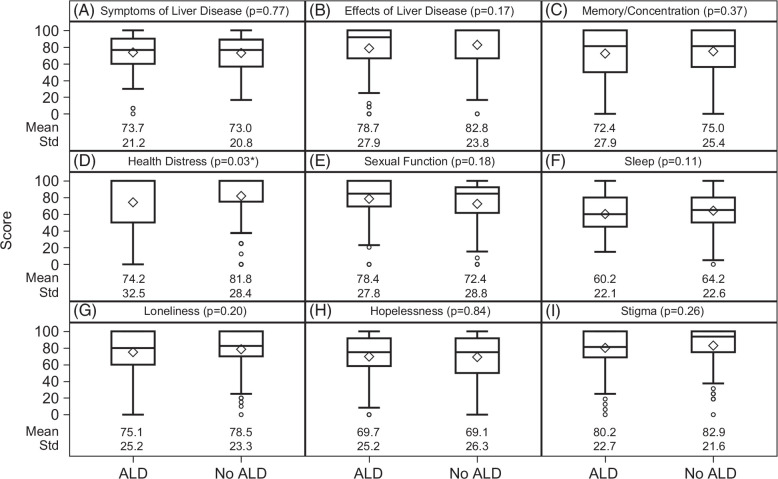
Boxplots are used to show the dispersion of the data, and the mean is represented by the diamond symbol. In addition, the numerical values of means and standard deviations are presented for each pair of comparison. Cirrhosis etiology was not statistically associated with most LDQoL subscale scores (A-C, E-I), except for health distress (D), where patients with ALD etiology had lower LDQoL health distress subscale scores than patients with non-ALD etiology. *p<0.05. Abbreviations: ALD, alcohol-associated liver disease; LDQoL, liver disease quality of life.

**FIGURE 3:**
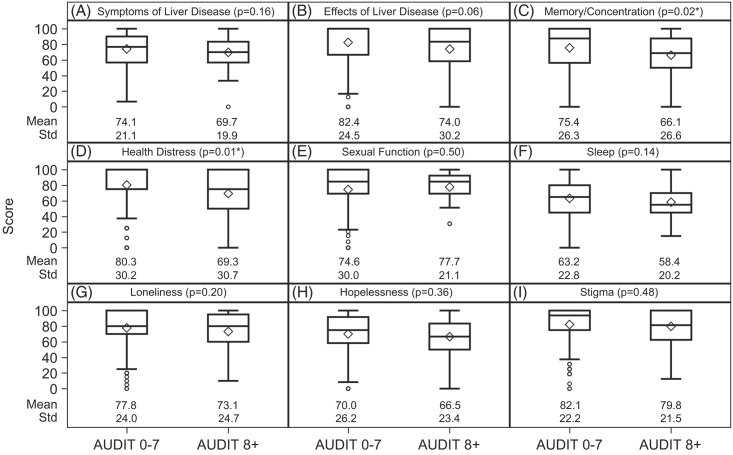
Boxplots are used to show the dispersion of the data, and the mean is represented by the diamond symbol. In addition, the numerical values of means and standard deviations are presented for each pair of comparison. Problematic drinking was associated with lower LDQoL memory/concentration (C) and health distress (D) subscale scores, but was not statistically associated with the other LDQoL subscale scores (A-B, E-I). *p<0.05. Abbreviations: AUDIT, Alcohol Use Disorders Identification Test; LDQoL, liver disease quality of life.

The mean overall LDQoL scores did not statistically differ between the non-ALD group (M=75.6, SD=16.7) and the ALD group (M=73.1, SD=18.1, *p*=0.21). The mean overall LDQoL score was lower in the AUDIT ≥8 group (M=69.8, SD=17.4) than the AUDIT <8 group (M=75.4, SD=17.2, *p*=0.03). Analyses of the LDQoL subscales are shown in Figure 2 (by cirrhosis etiology) and Figure 3 (by problematic drinking). Compared with the non-ALD group (M=81.8, SD=28.4), the ALD group had worse health distress QoL (M=74.2, SD=32.5, *p*=0.03; Figure 2D), reflecting more health distress concerns. Two LDQoL subscale scores differed significantly by the AUDIT clinical threshold score of 8. Compared with the AUDIT <8 group (M=75.4, SD=26.3), the AUDIT ≥8 group had worse memory/concentration QoL (M=66.1, SD=26.6, *p*=0.02; Figure 3C). Compared with the AUDIT <8 group (M=80.3, SD=30.2), the AUDIT ≥8 group also had worse health distress QoL (M=69.3, SD=30.7, *p*=0.01; Figure 3D).

In multivariable general linear model adjusting for age, sex, race/ethnicity, site, pandemic-related stress, and history of anxiety or depressive disorder, the associations of problematic drinking with LDQoL overall, memory/concentration, and health distress scores were statistically significant (Table 2). Across these 3 models, higher pandemic-related stress was associated with worse LDQoL scores. For the LDQoL overall scale, participants with other race/ethnicity reported better LDQoL scores than White participants (*p*=0.03), whereas history of anxiety or depressive disorder was associated with worse overall LDQoL scores (*p*=0.02). For the LDQoL health distress subscale, younger age was associated with worse health distress QoL (*p*=0.004). Sensitivity analyses with additional statistical adjustment for liver disease severity (MELD-Na as well as history of past or current liver decompensation) were conducted (Table 3). For the overall LDQoL scale, current decompensated cirrhosis (vs. none) was associated with a 5.91-point decrease in overall LDQoL scores (95% CI: [−11.07, −0.75], *p*=0.025), and problematic drinking was associated with a 5.71-point decrease in overall LDQoL scores (95% CI: [−10.69, −0.72], *p*=0.025). For the 2 LDQoL subscales, problematic drinking remained significantly associated with LDQoL health distress scores (*b*=−8.77, 95% CI: [−17.50, −0.04], *p*=0.049) and LDQoL memory/concentration scores (*b*=−9.79, 95% CI: [−17.86, −1.71], *p* = 0.018).

**TABLE 2:** Multivariable generalized linear regression modeling effects of problematic drinking on LDQoL (N=329)

	Overall LDQoL		LDQoL Health distress		LDQoL Memory/concentration	
	*b* (95% CI)	*p*	*b* (95% CI)	*p*	*b* (95% CI)	*p*
Problematic drinking						
AUDIT≥8 vs.<8	−5.49 (−10.32, −0.66)	0.03	−8.89 (−17.45, −0.34)	0.04	−9.02 (−16.76, −1.28)	0.02
Age (y)	0.12 (−0.08, 0.32)	0.24	0.52 (0.17, 0.88)	0.004	−0.01 (−0.33, 0.30)	0.93
Sex						
Female vs. male	−4.40 (−9.89, 1.08)	0.12	−7.87 (−17.59, 1.84)	0.11	−0.16 (−8.95, 8.64)	0.97
Race/ethnicity						
Latino vs. White	3.49 (−1.03, 8.01)	0.13	4.15 (−3.86, 12.15)	0.31	−0.97 (−8.21, 6.28)	0.79
Other vs. White	5.34 (0.66, 10.02)	0.03	6.59 (−1.71, 14.88)	0.12	4.05 (−3.45, 11.55)	0.29
Site						
PAVA vs. ZSFG	−4.74 (−9.89, 0.41)	0.07	−8.77 (−17.88, 0.35)	0.06	−7.59 (−15.84, 0.66)	0.07
SFVA vs. ZSFG	1.36 (−4.26, 6.98)	0.63	2.03 (−7.93, 11.99)	0.69	−2.68 (−11.68, 6.32)	0.56
Pandemic-related stress (points)	−3.57 (−4.96, −2.18)	<0.001	−5.47 (−7.94, −3.01)	<0.001	−3.89 (−6.12, −1.66)	<0.001
History of anxiety or depressive disorder	−5.22 (−9.47, −0.98)	0.02	−3.91 (−11.43, 3.62)	0.31	−4.96 (−11.76, 1.85)	0.15

*Note:* Higher LDQoL scores reflect better QoL. Lower LDQoL scores reflect worse QoL.

Abbreviations: AUDIT, Alcohol Use Disorders Identification Test; LDQoL, liver disease quality of life; PAVA, Palo Alto Veterans Affairs Health Care System; SFVA, San Francisco Veterans Affairs Health Care System; ZSFG, Zuckerberg San Francisco General Hospital.

**TABLE 3:** Sensitivity analyses with statistical adjustment for liver disease severity on the associations between problematic drinking and LDQoL scores (n=310)

	Overall LDQoL		LDQoL Health distress		LDQoL Memory/concentration	
	*b* (95% CI)	*p*	*b* (95% CI)	*p*	*b* (95% CI)	*p*
Problematic drinking						
AUDIT≥8 vs.<8	−5.71 (−10.69, −0.72)	0.03	−8.77 (−17.50, −0.04)	0.049	−9.79 (−17.86, −1.71)	0.02
Age (y)	0.07 (−0.14, 0.27)	0.54	0.39 (0.02, 0.75)	0.04	−0.03 (−0.37, 0.31)	0.85
Sex						
Female vs. male	−5.36 (−11.00, 0.28)	0.06	−10.55 (−20.42, −0.68)	0.04	−0.71 (−9.84, 8.43)	0.88
Race/ethnicity						
Latino vs. White	3.36 (−1.28, 8.00)	0.16	5.31 (−2.81, 13.44)	0.20	−0.83 (−8.35, 6.69)	0.83
Other vs. White	4.46 (−0.34, 9.27)	0.07	5.98 (−2.44, 14.40)	0.16	3.40 (−4.38, 11.18)	0.39
Site						
PAVA vs. ZSFG	−5.91 (−11.29, −0.53)	0.03	−10.66 (−20.08, −1.24)	0.03	−8.84 (−17.56, −0.12)	0.047
SFVA vs. ZSFG	0.68 (−5.22, 6.59)	0.82	0.57 (−9.78, 10.92)	0.91	−3.71 (13.27, 5.86)	0.45
Pandemic-related stress (points)	−3.52 (−4.97, −2.07)	<0.001	−5.74 (−8.27, −3.20)	<0.001	−3.49 (−5.83, −1.14)	0.004
History of anxiety or depressive disorder	−5.07 (−9.38, −0.76)	0.02	−3.37 (−10.92, 4.18)	0.38	−4.34 (−11.33, 2.64)	0.22
MELD-Na Score	−0.27 (−0.70, 0.17)	0.23	−0.46 (−1.23, 0.30)	0.23	0.24 (−0.46, 0.94)	0.50
Decompensated cirrhosis						
Current	−5.91 (−11.07, −0.75)	0.03	−9.82 (−18.85, −0.79)	0.03	−3.97 (−12.33, 4.38)	0.35
Prior	−2.93 (−8.01, 2.15)	0.26	−14.10 (−22.99, −5.21)	0.002	−3.84 (−12.07, 4.38)	0.36

*Note:* Higher LDQoL scores reflect better QoL. Lower LDQoL scores reflect worse QoL.

Abbreviations: AUDIT, Alcohol Use Disorders Identification Test; LDQoL, liver disease quality of life; MELD-Na, Model for End-Stage Liver Disease-Sodium; PAVA, Palo Alto Veterans Affairs Health Care System; SFVA, San Francisco Veterans Affairs Health Care System; ZSFG, Zuckerberg San Francisco General Hospital.

## DISCUSSION

In this large and diverse sample of patients with cirrhosis, problematic drinking (scoring ≥8 on the AUDIT) was associated with worse liver disease QoL even after controlling for potential confounding factors including liver disease severity, particularly in the QoL domains of memory/concentration and health distress. These findings corroborate prior studies that have documented associations between reductions in alcohol drinking level and improvements in various domains of QoL among patients with AUD seeking treatment.^31,32^ Extending prior findings, the current study showed that the detrimental effect of problematic drinking on liver disease QoL was observed even among patients who had already developed cirrhosis. As the minimal clinically important difference for Short Form LDQOL is 5.1,^23^ changes in liver disease QOL from both problematic drinking (5.7) and liver decompensation (5.9) were clinically meaningful. Within the context of the COVID-19 pandemic, pandemic-related stress was consistently associated with worse liver disease QoL, highlighting the need for holistic treatment to address QoL and well-being concerns among diverse populations.^20^

Clinically, total abstinence from alcohol is the recommendation for patients with chronic liver disease, and especially for those with cirrhosis.^33^ Effective management of patients with chronic liver disease requires detection of alcohol use, evaluation of alcohol use patterns and alcohol-associated problems, and provision of alcohol treatment for those who need it.^34^ Yet a large percentage of patients with ALD struggle to stop drinking and remain abstinent. In the current study, 51% of those with ALD reported any alcohol use in the past year and 35% had a score of 8 or higher on the AUDIT. As ALD etiology was not significantly associated with worse overall liver disease QoL, alcohol-associated cirrhosis etiology may represent past rather than ongoing alcohol use. Moreover, even among those with non-ALD etiology, 30% reported any alcohol use in the past year, suggesting an unmet alcohol treatment gap in the management of non-ALD cirrhosis. Together, these findings highlight the utility of screening for current alcohol use and related harms using validated tools such as the AUDIT. Optimally, integrated hepatology care using a multidisciplinary team approach can link patients who are at risk for poor outcomes to tailored treatments that simultaneously target AUD and its associated liver disease.^35^

In our analyses using LDQoL subscales, worse memory/concentration QoL was found among those who reported problematic drinking than those without even when adjusting for severity of liver disease. Cognitive deficits among individuals with AUD are evident across multiple neurocognitive domains, including impaired working memory and executive functioning.^36^ Deficits in executive function and attentional control are associated with more years of alcohol consumption and higher alcohol craving.^37^ The addiction cycle is a validated model describing how impulsivity and compulsivity drive substance use behaviors that can be characterized into 3 stages, including binge/intoxication, withdrawal/negative affective, and preoccupation/anticipation.^38^ Conceptually, deficits in memory/concentration among those with AUD would map onto alcohol craving and executive dysfunction associated with the preoccupation/anticipation stage within the addiction cycle. While abstinence from alcohol can lead to improved cognitive functioning among some individuals in recovery, many individuals with problematic drinking continue to exhibit significant cognitive impairment even after prolonged sobriety.^39^ This may be especially true for patients with cirrhosis, who often have either overt or subclinical hepatic encephalopathy,^40^ and for older individuals who use alcohol, as both aging and alcohol misuse contribute to cognitive decline.^41^ Accordingly, clinicians could assess whether memory-related and concentration-related challenges are present among individuals with comorbid AUD-ALD. If so, highlighting how alcohol-associated cognitive deficits could compromise their QoL and put them at risk for severe outcomes such as dementia, falls, and other injuries could potentially be used to help motivate abstinence.

The richness of the multidimensional Short Form LDQoL instrument allowed us to gain a nuanced understanding of the associations between problematic drinking and specific types of distress. Notably, problematic drinking was not significantly associated with LDQoL domains related to symptoms or effects of liver disease, loneliness, or hopelessness, but it was associated with lower health distress QoL. The health distress subscale of the Short Form LDQOL assessed for frustration and feeling of weighed down that are specifically attributed to the liver disease. Together, results indicate that individuals who scored ≥8 on the AUDIT may not necessarily have had worse liver disease symptoms or feel more lonely or hopeless than individuals who scored <8 on the AUDIT, but the level of distress caused by their liver disease still was subjectively higher. This novel finding highlights the need to help individuals with problematic drinking better manage psychological distress associated with their drinking and liver disease. Using alcohol to cope with liver disease–specific negative emotions may be a mechanism through which individuals with comorbid AUD-ALD are kept within the addiction cycle. As alcohol is often consumed to attain relief from physical symptoms and anxiety,^42^ more research is needed to understand if worse health distress QoL among those with problematic drinking can be targeted using behavioral alcohol interventions such as cognitive behavioral and motivational enhancement therapies.^43^

There is some conceptual overlap between alcohol-specific and liver disease–specific QoL, which may benefit from further investigation. For example, a sleep QoL domain can be found in both the LDQoL instrument and the Alcohol QoL Scale.^44^ The role of stigma may impede health care access for individuals with AUD and ALD.^45,46^ Although these QoL domains did not vary based on the AUDIT clinical threshold, concerns about sleep and stigma QoL are likely present among some patients with AUD and ALD. Among patients with ALD, factors such as drinking severity, self-stigma, and self-efficacy were positively associated with increased motivation to change.^47^ Moving forward, an important future direction is to understand how individuals with ALD may be reached at earlier stages of disease progression, such as through brief mobile health interventions to increase awareness, motivation, or alcohol treatment engagement, and to minimize the adverse impact of problematic drinking and ALD on QoL.

This study has several unique strengths, including a large sample based on multicenter data collection, the use of detailed and validated measures of problematic drinking and liver disease QoL, and a diverse clinical sample of socioeconomically and medically disadvantaged patients. Despite these strengths, this study has several limitations. First, inherent to survey-based studies, recall bias and response bias may be present, and social desirability may have led to underreporting of alcohol use and related problems. Second, the sample was recruited from liver clinics from health systems in Northern California, including 2 VA health care systems that had higher proportions of men. Thus, findings may not be generalizable to individuals who are not served by these health systems, privately insured patients, or those who reside in other states. Third, the Patient Reported Outcomes Measurement Information System-29 (PROMIS-29) has recently been proposed as the superior instrument for health-related QoL research among patients with cirrhosis given its validity, ease of use, and absence of floor or ceiling effects.^48,49^ Nevertheless, the validated Short Form LDQoL chosen for this study was considered appropriate for our research questions as it included both liver-specific and general QoL domains.^21,23,50^ Finally, cross-sectional data were analyzed, and so the direction of effects could not be ascertained. For instance, while problematic drinking may negatively impact LDQoL, low LDQoL may also influence drinking behavior. Future research can use a longitudinal design to examine potential bidirectional associations between problematic drinking and LDQoL domains over time.

## CONCLUSIONS

In this multicenter study of patients with cirrhosis, we found associations between problematic drinking and worse liver disease QoL, especially in the QoL domains of memory/concentration and health distress. These associations remained significant after statistical adjustment for potential confounding factors such as pandemic-related stress and severity of liver disease including decompensated cirrhosis status. The consideration of QoL factors among patients with late-stage liver disease, and particularly those with ALD, is consistent with the call to examine QoL in addiction treatment and the recognition by NIAAA to include measures of QoL as an integral part of recovery from AUD.^16^ Increased emphasis on the assessment of QoL within integrated liver care can help clinicians to identify deficits in executive functioning and problems with managing liver disease–specific negative emotions that can perpetuate the addiction cycle. To address these clinical factors that can underlie AUD, the utilization of evidence-based treatment approaches to manage negative symptoms and enhance QoL is needed. Novel intervention paradigms such as telehealth approaches to motivational enhancement and provision of AUD treatment in liver disease care settings should be developed and tested in future research.
